# Discovery and Therapeutic Targeting of Differentiated Biofilm Subpopulations

**DOI:** 10.3389/fmicb.2019.01908

**Published:** 2019-08-27

**Authors:** Karishma Bisht, Catherine Ann Wakeman

**Affiliations:** Department of Biological Sciences, Texas Tech University, Lubbock, TX, United States

**Keywords:** biofilm, heterogeneity, infectious disease, technology, antibiotic resistance, subpopulations, therapeutic targeting

## Abstract

The association of microorganisms into biofilms produces functionally organized microbial structures that promote community survival in a wide range of environments. Much like when individual cells within a multicellular organism express different genes from the same DNA blueprint, individual microbial cells located within different regions of a biofilm structure can exhibit distinct genetic programs. These spatially defined regions of physiologically differentiated cells are reminiscent of the role of tissues in multicellular organisms, with specific subpopulations in the microbial community serving defined roles to promote the overall health of the biofilm. The functions of these subpopulations are quite diverse and can range from dormant cells that can withstand antibiotic onslaughts to cells actively producing extracellular polymeric substances providing integrity to the entire community. The purpose of this review is to discuss the diverse roles of subpopulations in the stability and function of clonal biofilms, the methods for studying these subpopulations, and the ways these subpopulations can potentially be exploited for therapeutic intervention.

## Introduction

The bulk of microbiological studies in history have focused on the study of planktonic, freely floating microorganisms. However, work over the past several decades has demonstrated the importance of surface-adhered states of many microbes, such as those first described by Antonie van Leeuwenhoek back in the 17th century when he observed microorganisms present as aggregates on his dental plaque (Leewenhoeck, 1684). It is now known that communities of microorganisms exist as aggregates embedded in a self-produced matrix made up of extracellular polymeric substances (EPS). The EPS matrix encasing this community consists of polysaccharides, lipids, proteins, and/or DNA. J. W. Costerton, a founding father in the research of these microbial communities, described this structure as a biofilm (Costerton et al., 1999).

These microbial structures have sparked a lot of interest in the last three decades, as it has become clear that biofilm formation may be the preferred bacterial lifestyle in nature (Moons et al., 2009). Biofilms can be found in virtually every natural and man-made environment and therefore significantly impact human health and industry (Hall-Stoodley et al., 2004). The diverse niches occupied by biofilms include the bottoms of streams or river beds as well as the surfaces of stagnant pools of water in which these communities play an important role in the aquatic food chain (Battin et al., 2016). These microbial communities are also highly associated with the human body, often serving benign or beneficial roles and sometimes providing reservoirs for pathogenic bacteria (Parsek and Singh, 2003; Gutt et al., 2018).

Pathogenic microorganisms employ versatile strategies to invade the human body and evade the host immune system, including biofilm formation. It is the unique architecture of biofilms, which includes the EPS matrix and the cells within, that enables these microbial structures to persist in a wide range of environments, including the host environment (Costerton et al., 2003; Tseng et al., 2018). Biofilms can form from cultures containing a single microbial species or from numerous and diverse types of microorganisms (Peters et al., 2012). The multicellular, often multispecies, and even multi-kingdom composition of biofilms results in the development of specialized adaptations, making these structures extremely recalcitrant against antibiotics and difficult to act on by the host defenses like phagocytosis (Berit et al., 2002; Harriott and Noverr, 2010; De La Fuente-Núñez et al., 2013; Baishya and Wakeman, 2019). The antibiotic tolerance of biofilms is partially imparted by the limited diffusion of antibiotics through the biomass due to both the presence of EPS and cell density (Mah and O’toole, 2001; Goltermann and Tolker-Nielsen, 2017). Also, the presence of physiologically differentiated distinct subpopulations within these multicellular communities contributes to antimicrobial tolerance (Høiby et al., 2010). Due to many antibiotics targeting specific physiological processes, subpopulations repressing these processes (such as metabolically dormant populations residing deep within anoxic regions of the biomass) are intrinsically resistant to certain antibiotics (Pamp et al., 2008). Therefore, understanding biofilm architecture, identifying the role of biofilm subpopulations in maintaining the integrity of these communities, and discovering weaknesses in the “biofilm armor” are crucial to human health.

## Biofilms as Heterogeneous Populations

### Biofilm Subpopulations Result in Increased Antibiotic Tolerance

**FIGURE 1:**
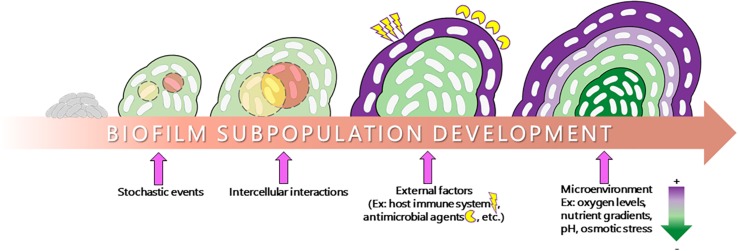
Schematic representation of subpopulation development in a biofilm. The transition from a planktonic lifestyle to a surface-adhered biofilm lifestyle involves a number of factors. Stochastic events such as spontaneous mutation, external stimuli such as host immune factors and antibiotic treatment, intercellular interactions, and internal diffusion of oxygen, nutrient, and various stressors play a major role in influencing the formation of microenvironments and the physiology of the biofilm, resulting in the formation of distinct subpopulations within the biofilm strata (depicted as different colors within the biomass).

Bacteria within biofilms can survive in different environmental niches owing to their distinct cell physiology (Stewart and Franklin, 2008; Nadell et al., 2016). The physiology occurring within biofilms is quite complex, with different cell populations exhibiting entirely different gene expression and metabolic profiles, even within a biofilm derived from clonal populations that possess the same set of genetic material. This ability to differentiate into a diverse array of cell types can be attributed to a number of internal and external factors influencing microbial gene expression (Figure 1). Indeed, these metabolic changes can be due to stochastic factors such as spontaneous mutation, which fundamentally changes the genetic material within a cell. However, a large amount of this differentiation requires no such events and instead is directly influenced by stimuli specific to the microenvironments within the biomass. These microenvironments can be produced by differential diffusion of intercellular signaling molecules, external stressors, nutrient/oxygen, and waste products (Stewart and Franklin, 2008; Monds and O’toole, 2009; Zheng et al., 2015). The formation of microenvironments creates a feedback loop in which the microenvironments of a biofilm drive changes in microbial physiology and the different physiologies drive the formation of microenvironments, which results in a large amount of phenotypic diversity within the biomass. As the biomass thickens, microenvironments become more pronounced and larger numbers of subpopulations emerge. The function of these subpopulations within the communities as well as the discovery and therapeutic targeting potential surrounding these subpopulations is the focus of this review.

Various studies have focused on the understanding of these heterogeneous subpopulations of cells within a biofilm stratum, which are characterized by distinct genetic programs, spatial segregation, and differential antibiotic susceptibility (Haagensen et al., 2007; Pamp et al., 2008; Yan and Bassler, 2019). Due to the number of pathogens capable of forming biofilms during infection, the role of phenotypic heterogeneity in the formation of antibiotic-resistant populations is of particular interest. Previous work has reported the presence of distinct subpopulations in *Pseudomonas aeruginosa* biofilm, mainly in the stalk and cap region of these mushroom-shaped multicellular structures imparting unique antibiotic-tolerant properties to these regions (Bjarnsholt et al., 2005; Banin et al., 2006; Haagensen et al., 2007; Kaneko et al., 2007; Pamp et al., 2008). Another example of antibiotic tolerance due to subpopulations can be observed in *Escherichia coli* biofilm. Upon treatment with ampicillin, the subpopulations in the deeper layers remain resistant while the young colony biofilm is susceptible to ampicillin (Ito et al., 2009). Increased antibiotic tolerance observed within the depths of a biomass can likely be attributed to a combination of limited antibiotic diffusion as well as the altered microbial physiologies occurring within this microenvironment.

Under nutrient-limited conditions, bacteria in a biofilm reduce the production of metabolites and increase antioxidant defenses resulting in antibiotic tolerance (Nguyen et al., 2011). These slow-growing biofilm subpopulations can withstand multiple classes of antibiotics. For example, the dormant, slow-growing subpopulation found deep within the biofilm biomass of *P. aeruginosa* are known to be more tolerant to tobramycin and ciprofloxacin in comparison to the outer metabolically active subpopulation (Williamson et al., 2012). A recent study highlighted the existence of metabolic heterogeneity in the hypoxic region of biofilm subpopulations of *P. aeruginosa* and its effect on both the metabolism and antibiotic tolerance in bacteria. The presence of phenazine, a redox-active pigment, was responsible for this metabolic heterogeneity, which resulted in the cells being more tolerant to ciprofloxacin (Schiessl et al., 2019).

While most antibiotic-tolerant subpopulations that have currently been characterized are the metabolically dormant cells deep within the biomass, this is not always the case. A specific membrane-targeting antimicrobial like colistin was able to target the dormant inner population of biofilm while the metabolically active outer subpopulation was tolerant to this attack. The presence of unique physiological adaptations and regulation of specific genetic machinery in the active subpopulation was responsible for this behavior (Chiang et al., 2012).

This finding indicates that not all subpopulation-specific antibiotic tolerance is simply attributed to metabolic dormancy and, in actuality, is due in large part to the specific metabolic adaptations of distinct biofilm subpopulations.

### Additional Biofilm Subpopulations Participate in Shared Resource Production

While most microbial phenotypes are selfish traits, there is evidence that the distinct biofilm subpopulations can adopt more cooperative roles in the microbial community to promote the overall stability and integrity of the biofilm (Brockhurst et al., 2006; Michod and Herron, 2006). Both the selfish traits, such as subpopulations individually adapted to become more resistant to antibiotics, and more altruistic physiological adaptations, such as subpopulations engaged in the production of community resources, can contribute to increased tolerance to various environmental challenges.

The origin of the evolutionary transition between unicellular and multicellular forms has been debatable at different levels of selection (Axelrod and Hamilton, 1981; Rainey and Rainey, 2003; Michod, 2007). Importantly, bacterial subpopulations can theoretically benefit from being in a multicellular form as they not only can gain collective protection against antagonists but also participate in the division of labor to streamline biosynthetic energy costs (Shapiro, 1998; Kaiser, 2001). This strategy can enable survival of that population in certain harsh conditions. It has been theorized that other subpopulations also play more altruistic or community-centric roles in the development of multicellular communities through the division of labor (Gestel et al., 2015). This lesser-studied feature of biofilm subpopulations plays an important role in the formation of biofilm structures (Dragos et al., 2018). Through the division of labor, the seemingly altruistic act of producing a shared resource then becomes beneficial for both the producer and the entire community. This can be linked to the cells of a differentiated multicellular organism benefiting from cooperation mediated by sharing and producing nutrients with the other cells. Thus, cooperation is considered to be one of the important criteria for building a differentiated multicellular organism (Michod and Roze, 2001).

The heterogeneous development of biofilm in *Pseudomonas* involves mechanisms leading to complex subpopulation interaction (Williamson et al., 2012). One study on biofilm formation in *P. aeruginosa* discussed the role of subpopulation interactions in the formation of mushroom-shaped structures formed by *P. aeruginosa* biofilms (Yang et al., 2009). In addition to the differing antibiotic tolerance, it has been found that the different populations in these structures undergo a division of labor such that the synthesis of the iron-scavenging molecule pyoverdine produced by one subpopulation is used for the growth of another subpopulation, which itself does not express the pyoverdine synthesis genes (Yang et al., 2009). Additionally, the distribution of this molecule in biofilm subpopulations of *P. aeruginosa* was found to be more concentrated at the center of the colony. A quantitative explanation of the formation of this gradient was given by stating that it formed because of a local exchange between the contacting cells (producer and non-producer cells) and not by global diffusion of pyoverdine (Julou et al., 2013). Another type of division of labor was recently reported in *P. aeruginosa* biofilms where cyclic-di-GMP, a secondary messenger signaling molecule important for transitioning of cells from a planktonic to a biofilm lifestyle, was playing a major role in imparting heterogeneity to the clonal population on sensing a surface. The subpopulation with higher cyclic-di-GMP produced biofilm matrix while the other subpopulation having low cyclic-di-GMP was involved in surface motility (Armbruster et al., 2019). This type of division of labor within a microbial community ensures greater fitness of the cell population as a whole by enabling portions of the community to be protected within a biofilm while enabling other portions of the community to explore surfaces for resources.

This type of differentiation and division of labor exists in many microbial cell types. For example, the biofilm subpopulations of *Staphylococcus aureus* have been shown to be genetically identical but physiologically distinct due to different gradients, including oxygen gradients, within the biofilm strata. This distinct trajectory led to a number of different cell states including aerobically respiring cells, fermentative cells, dead cells, and dormant cells (Rani et al., 2007). These dormant subpopulations may be contributing to a “selfish” adaptation of antibiotic resistance. However, other subpopulations of *S. aureus* biofilms appear to be involved in shared resource production. For example, the presence of heterogeneous expression of cell-death-associated *cid* and *lrg* operons due to varied oxygen availability is responsible for differential expression of cell death and lysis within biofilm subpopulations of *S. aureus*. The study revealed the presence of distinct biofilm subpopulations where one subpopulation was releasing excessive eDNA and other cellular components due to more *cid* expression while the other subpopulation did not release any eDNA, thus exhibiting a distinct pattern of gene expression and physiological characteristics (Moormeier et al., 2014). Another important study showing heterogeneity in *S. aureus* populations is the *Agr* quorum-sensing system, which under varying environmental conditions, can be expressed in different subpopulations. The *S. aureus* subpopulation with an active *agr* system will result in bacterial dispersion, which is thought to contribute more to acute infections while the subpopulation with an inactive *agr* system will commit to biofilm lifestyles more associated with chronic infections (García-Betancur et al., 2017).

In *Bacillus subtilis*, another biofilm-forming organism, the biofilm matrix consists of two important structural components, namely, EPS, the exopolysaccharide, and TasA, a protein component of the matrix. Different subpopulations are involved in the production of biofilm matrix components with some subpopulations producing only the EPS while the others produce both EPS as well as TasA. Since these matrix components are costly to produce, they are shared by the cells reducing the overall metabolic costs for the clonal community (Dragos et al., 2018). Similarly, the cells of macrocolony biofilm of *E. coli* also goes through a division of labor, resulting in a heterogeneous production of extracellular matrix in the intermediate layer of the biofilm. Localization of different regulators in this layer results in the formation of matrix producers and non-producers and this local cellular heterogeneity was found to be important for the structural integrity of the biofilm (Serra and Hengge, 2019). Public goods are energetically costly to produce and provide a benefit to all the individuals in the vicinity. These studies represent only a subset of the excellent and ongoing work characterizing the heterogeneity in biofilms produced by a diverse array of microorganisms and serve as great examples showing the utilization of shared resources by neighboring subpopulations of a clonal microbial biofilm for its survival.

Production of shared resources associated with the division of labor can also select for non-cooperative individuals termed as cheaters. Cheaters are community defectors who exploit the cooperative acts and reap the benefit with no energetic cost of production. Cheating is thus the utilization of public goods that can reduce the overall productivity of the biofilm (Popat et al., 2012). Cheating behavior is common in many natural systems and there have been instances when non-cooperators can have a selective advantage over the cooperators. This can result in a stable mutualistic association where both the populations exist together or could lead to reciprocal extinction (Axelrod and Hamilton, 1981; Doebeli and Knowlton, 1998; Ferriere et al., 2002; Roberts and Renwick, 2003; Sachs et al., 2004). “Tragedy of Commons” is a great example to show the cooperator–cheater relationship as it is often associated with the over-exploitation of a common good by cheaters (West et al., 2006; Diggle et al., 2007). This phenomenon highlights the fine balance that must occur within microbial communities undergoing division of labor. Therefore, communities possess “policing” strategies to ensure that these cheaters are removed from the community (Yan et al., 2018).

The discovery of hundreds of differentially expressed gene products localizing to dozens of uncharacterized biofilm subpopulations of organisms such as *P. aeruginosa* grown under various conditions has been recently reported by different research groups (Lenz et al., 2008; Chua et al., 2016; Wakeman et al., 2016; Babin et al., 2017; Dunham et al., 2017). These subpopulations are highly reproducible and appear to be contained within the distinct microenvironments formed within a developing biofilm. The existence of these genetic programs indicates that they are likely to play a role in microbial survival under certain stress conditions experienced within the various environments capable of being colonized by the bacteria. Therefore, it is important to elucidate the functions of these newly discovered subpopulations and to develop tools enabling the discovery of novel biofilm subpopulations in other biofilm-forming pathogens.

### Emergent Technologies for the Study of Biofilm Subpopulations

The tools and technologies that researchers are applying to the study of biofilm subpopulations are ever evolving. Biofilms are complex and dynamic in nature, and therefore, to understand the activities occurring within these structures, numerous tools have been designed or adapted to characterize biofilms at the molecular, cellular, and systems levels. These techniques range from the use of mutagenesis, enzyme activity assays, and reporter gene fusions to the use of imaging and -omics approaches.

Many remarkable advances have occurred in biofilm research during the past few decades. Scanning electron microscopy is one important technique that has been used over decades along with other standard microbiological culture techniques for studying biofilms. The findings and results obtained from the research have opened our eyes to this understudied area of microbial biology (Priester et al., 2007). These techniques have not only helped researchers to characterize the ultrastructure of biofilms but also helped them to elucidate the genes involved in biofilm growth and development. It is now known that biofilms are not simply organisms growing and forming a slime layer on a surface but rather highly organized biological system where microbes form structured and functional communities (Davey and O’toole, 2000).

In addition to electron microscopy, various other imaging technologies have been employed by biofilm researchers. For example, fluorescence microscopy has been a powerful tool to advance our understanding of biofilm structure, development, and function. Confocal laser scanning microscopy (CLSM) is extensively used for in-depth analysis of the structure and composition of live biofilms (Berk et al., 2012; Colvin et al., 2012; Reichhardt and Parsek, 2019). By using modified fluorescent fusion proteins, fluorescent probes and stains, or fluorescently labeled antibodies, researchers have been able to interrogate biochemical environments in living cells in three dimensions. Another important molecular technique under this category that has been used for quite some time now is fluorescent *in situ* hybridization (FISH), which is used to detect the abundance of multiple bacterial species present in biofilm samples by hybridizing to the 16S rRNA or any specific sequence of nucleic acid (Lebeer et al., 2011). Recent advances to FISH using peptide nucleic acid (PNA) probes have been made. PNA FISH is a novel diagnostic technique that uses uncharged DNA analog (pseudopeptide) probes with higher specificity and has been used by researchers to study the spatial distribution of distinct subpopulations in a microbial community (Beebout et al., 2019).

These fluorescence studies can be strengthened through the use of supplemental techniques such as microfluidics combined with video microscopy. This combination of techniques has been used for controlled biofilm studies as it helps mimic the natural microbial habitats and therefore can be used to visualize the intricate processes associated with biofilm formation and its heterogeneous nature under different environmental conditions (Moormeier et al., 2013, 2014). Also, combining microelectrodes with the use of fluorescent reporters can reveal features of the biofilm microenvironments that drive the physiological differentiation within biofilms (Rasmussen and Lewandowski, 1998; Christen et al., 2010; Moya et al., 2014). Additional imaging advancements aiding in the discovery of biofilm heterogeneity include single-cell live imaging (Yan et al., 2016; Armbruster et al., 2019; Hartmann et al., 2019). For example, recent use of this type of imaging combined with the creation of a riboswitch-controlled fluorescent reporter revealed heterogeneous levels of cyclic di-GMP in *B. subtilis* subpopulations (Weiss et al., 2019).

While there are numerous scientific advances contributing to the strengths of imaging technologies for the study of biofilm structure, there are also inherent disadvantages to these techniques. One such disadvantage for certain types of labeling is that the use of chemicals to fix the probes prior to hybridization may disturb the structure of the biofilm and make time-course studies difficult (Amann and Ludwig, 2000). Moreover, imaging of small antibiotic molecules and other antimicrobials is problematic when using this technique as the addition of fluorescent tags could change the analytes’ activity and distribution (Garcia-Betancur et al., 2012). Another major limitation of any technique requiring fluorescent tagging is that the target protein must be known prior to studying it – the appropriate fluorescently labeled probes cannot be designed otherwise. Overall, the methods of fluorescent labeling are evolving and the microscopes used for biofilm analysis are improving, making fluorescence imaging one of the best ways to study biofilm heterogeneity.

Flow cytometry (FCM) is another important technique reliant on the ability to fluorescently label cell populations. FCM and fluorescence-activated cell sorting (FACS) have been classically employed by fields such as immunology to identify cell populations expressing different surface markers. However, it has been recently proposed that this technology could be exploited by biofilm researchers to enable the analysis of subpopulations of bacterial cells with different physiological states such as those arising within biofilms (Ambriz-Avina et al., 2014). Once these populations have been labeled and separated out via FACS, they can be independently characterized by high-throughput molecular techniques (Trip et al., 2011) and studied using proteomic or transcriptomic analyses (Ambriz-Avina et al., 2014). Recently, FCM has been used for studying the distribution of different subpopulations present in the biofilm strata under different nutrient availability and at different stages of biofilm formation (Wojciech et al., 2018). Ultimately, this approach suffers from the same weakness as other fluorescent techniques in that a target for labeling subpopulations must be chosen, which limits the discovery of new subpopulations. However, the strength of studying subpopulations via FACS is that once these populations are separated out from the remainder of the biomass and have been studied via transcriptomics and proteomics, the fundamental physiology driving this differentiation can be elucidated and the functions of these populations can be better predicted.

Similarly, the use of laser capture microdissection (LCM) enables the study of localized biofilm processes when used in combination with transcriptomics or proteomics. This approach involves the isolation and capturing of subpopulations of bacteria from different regions of the biofilm for molecular analysis. LCM has a number of advantages as only the cell or subpopulation captured is retained and held for molecular analysis, thereby avoiding the other cellular debris (Lenz et al., 2008). Individual gene analysis can then be done by using RT-qPCR for the RNA extracted from captured cells (Lenz et al., 2008; Perez-Osorio et al., 2010). Alternatively, the RNA may be amplified and used for transcriptomic analysis (Williamson et al., 2012). This approach has excellent sensitivity and can be used for quantitative study of gene expression from distinct regions within the biofilm strata. Transcriptomics can be utilized to study genes that are differentially regulated at different layers within the biofilm architecture. It helps in elucidating the expression and function of various unknown genes, which are critical for biofilm formation (Heacock-Kang et al., 2017).

While both transcriptomic and proteomic analyses have provided pivotal pieces of information regarding biofilm heterogeneity, recent advances in proteomic studies and other mass-spectrometry-based techniques have been particularly successful. One such technique that has become increasingly popular among biofilm researchers is matrix assisted laser desorption/ionization imaging mass spectrometry (MALDI-IMS). This technology was first developed to visualize the heterogeneity of proteins and small molecules within the tissues of multicellular organisms but has also been particularly effective in the study of microbial communities (Caprioli et al., 1997; Moore et al., 2014). Any prior knowledge of the molecular targets of interest or any molecular tagging mechanism is not required when using MALDI-IMS, making this technique superior to other existing technologies for the study of biofilm heterogeneity (Wakeman et al., 2016). It has been extremely useful for the study of many microbial communities especially in understanding cellular heterogeneity, intercellular communication and the dynamics of single and multispecies microbial communities (Dunham et al., 2017). Researchers have also used this technique to uncover the stratified subpopulations in a pellicle biofilm, a biofilm that is formed at the air–liquid interface (Floyd et al., 2015). While the advantage of using this method is the identification of differentially expressed protein with no prior knowledge of which proteins might be differentially expressed, this technology is also associated with inherent weaknesses. For example, protein identification can be problematic due to the protein size not matching predicted sizes because of modifications and processing events. Also, there is a major size limitation where only proteins less than approximately 25 kDa can be visualized and only the most abundant proteins can be detected.

Other useful mass spectrometry (MS)-based quantitative proteomics approaches to characterize the physiologies of sensitive and antibiotic-tolerant subpopulations in biofilms are tools such as pSILAC and BONCAT. The pSILAC technique involves the labeling of amino acids of the biofilm cells with a stable isotope, which are then exposed to a stressor like antibiotics. The newly expressed protein in the biofilm cells that has now adapted to the antibiotic exposure is then quantified under pulse and no pulse conditions. This technique thus enhances our understanding of biofilm heterogeneity and studying subpopulation-specific response to antibiotics (Chua et al., 2016). A chemical biology method has also been used to identify proteins in a distinct subpopulation of interest in a biofilm on antibiotic exposure. The researchers used bio-orthogonal non-canonical amino acid tagging (BONCAT) to study proteome dynamics of phenotypically distinct subpopulations. In this technique, specialized tRNA synthases are genetically engineered to be differentially expressed within certain subpopulations. These tRNA synthases enable the incorporation of non-canonical amino acids within the labeled subpopulation, which allows for the identification of specific subpopulation proteome relative to the rest of the biofilm proteome. While this technology also requires prior tagging of a defined subpopulation, it can identify differentially expressed proteins of low abundance easily (Babin et al., 2017).

In addition to using technologies to elucidate the physiology of the cells within the biofilm, new technologies are being developed to enable the study of the structural features that lead to the formation of microenvironments and subpopulations within biofilms. For example, time of flight–secondary-ion mass spectrometry (TOF-SIMS) is a potentially powerful tool for depth profiling analysis and can help us better understand the diffusion of antimicrobials in biofilms (Davies et al., 2017). This technique can be used to image compounds present in both the exterior and interior of biofilm, thereby giving the complete information of the diffusion of these molecules throughout the biomass. Additionally, a high-resolution optical imaging technique called white-light interferometry (WLI) has been used to study the structural dynamics in bacterial biofilms. It is a non-destructive imaging method that makes use of a microfluidic flow cell to observe transitions in live biofilms using WLI. The structural changes in a mature biofilm and their response to external stressors like antibiotics can be studied using this method (Brann et al., 2017). Finally, X-ray micro-computed tomography (μCT) is another imaging technique used to study the mineralized areas within biofilms. The mineralization was a result of the accumulation of calcium carbonate within the bacterial biofilm. The development of an effective method to study the physiological role of mineral deposits within biofilm can help predict the rate of antibiotic penetration and the success of antibiotic treatment. In the long run, this X-ray technology could be used to image biofilms in medically relevant settings and also give us information on the diffusion rate of antibiotics within the biofilm (Keren-Paz et al., 2018).

**FIGURE 2:**
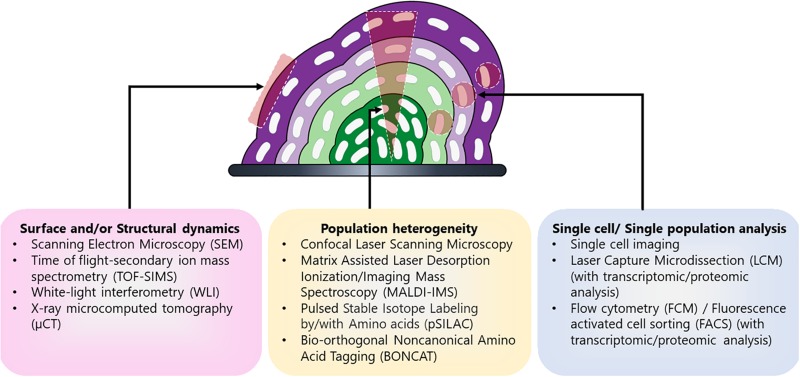
Different techniques used for detailed characterization of distinct biofilm components. Biofilm is a complex community encompassing different subpopulations (depicted in shades of purple and green) and therefore multiple techniques have been employed to characterize the different aspects of biofilm including its formation, development, and heterogeneity. Different approaches are used for evaluating particular aspects of biofilm such as surface and/or structural dynamics of biofilms (e.g., surface architecture, EPS composition, or diffusion rates), the metabolic heterogeneity within the entire population, or in-depth physiological profiling of select cells and/or subpopulations within the biomass.

Each of these techniques has their advantages and disadvantages (Table 1) and is useful for elucidating distinct components of biofilm heterogeneity including surface and/or structural dynamics, metabolic heterogeneity, and in-depth physiological profiling of individual subpopulations (Figure 2). Therefore, studies employing a combination of techniques are likely to have the greatest success for the discovery of novel subpopulations and the elucidation of their function. By combining these and other new techniques, it is now possible to gain insight into the heterogeneous subpopulations present in these complex microbial communities. Insights into potential therapeutic targeting of these differentiated cells can be revealed by elucidating the function and physiology of these novel subpopulations.

**TABLE 1:** Advantages and disadvantages of the emergent technologies used to study biofilm subpopulations.

**Technique**	**Strength**	**Weakness**	**References**
**Microscopy and/or fluorescence based:**			
Fluorescence microscopy – Single cell live imaging	Real-time detection of spatial heterogeneity within the biofilm environment	The user must first know what targets they want to visualize and design methods to fluorescently tag these targets	Hartmann et al., 2019; Weiss et al., 2019
Flow cytometry/Fluorescence-activated cell sorting	Enables detection and isolation of heterogeneous populations when combined with proteomic or transcriptomic techniques	Requires some prior knowledge of the subpopulation of interest in order to design fluorescent labels	Trip et al., 2011; Ambriz-Avina et al., 2014; Wojciech et al., 2018
Laser capture microdissection	Excellent sensitivity and large dynamic range for studying biofilm subpopulation when combined with techniques such as transcriptomics	Not compatible with live cell analysis	Perez-Osorio et al., 2010; Williamson et al., 2012; Heacock-Kang et al., 2017
White-light interferometry	Can survey a large area with a single scan without sacrificing desired resolution. Can use living samples under wet conditions and without the use of labeling	Cannot resolve the overhangs in mushroom shaped biofilm	Brann et al., 2017
X-ray micro-computed tomography	High resolution, fast, and non-destructive	Signal-to-noise ratio is high, due to which the image quality is not clear	Keren-Paz et al., 2018
**Mass spectrometry based:**			
Matrix-assisted laser desorption/ionization (MALDI) imaging mass spectrometry	Does not require prior knowledge of the molecular targets of interest or any molecular tagging mechanism	Can only visualize small, highly abundant proteins	Caprioli et al., 1997; Moore et al., 2014; Floyd et al., 2015; Wakeman et al., 2016; Dunham et al., 2017
pSILAC and bio-orthogonal non-canonical amino acid tagging (BONCAT)	Allow for analysis of newly synthesized proteins in a high background of pre-existing proteins in a heterogeneous biofilm subpopulation	Low temporal resolution and compromised quantitative accuracy	Chua et al., 2016; Babin et al., 2017
Time of flight-secondary ion mass spectrometry	High spatial resolution and sensitivity and can perform depth profiling analysis	Surface sensitive, can have narrow range of surface detection limits	Davies et al., 2017

### Potential for Therapeutic Targeting of Biofilm Subpopulations

Biofilms are particularly devastating during infection because their structural adaptations are resistant to environmental stressors – biofilm-associated microbes are less likely to be cleared by our immune cells and they are more tolerant to antimicrobial therapies than non-biofilm-associated microbes (Ciofu and Tolker-Nielsen, 2019). It has therefore become a challenge for researchers and clinicians today to eradicate biofilms using anti-biofilm drugs as the antibiotic-driven treatments often fail to completely suppress the infection, despite high dosage and long-term treatment. Immediate measures are required for the development of anti-biofilm drugs to fully cure biofilm-related infections (Bjarnsholt et al., 2013). Here, we discuss the potential for exploiting distinct subpopulation level traits existing within the biofilm community as potential therapeutic targets.

In most cases of chronic infections, the pathogenic bacteria can enter a metabolically inactive or dormant state by forming persister cells (Lewis, 2010). A persister cell is a slow-growing biofilm-associated cell that is more tolerant toward multiple classes of antibiotics compared to their metabolically active counterparts (Balaban et al., 2019). Most of the current antibiotics available today target the actively growing bacterial cells and therefore identification of molecules targeting dormant and/or persister cell populations might provide a solution to treat biofilm-associated infections. The toxin–antitoxin modules have been shown to play a major role in persister formation and have therefore been proposed as potential targets for persister cells (Kaspy et al., 2013). However, their role leading to persistence has recently been called into question (Ronneau and Helaine, 2019). Additionally, studies performed in both *E. coli* and *S. aureus* highlight the role of adenosine triphosphate (ATP) in imparting antibiotic tolerance and persister formation in these biofilm-associated pathogens. Low cellular ATP levels can decrease the target activity of the antibiotics, thereby leading to persister formation (Conlon et al., 2016; Shan et al., 2017). Therefore, a metabolism-based strategy has been used by researchers for eradicating bacterial persisters by the generation of a proton-motive force (PMF) that increases the metabolic activity and facilitates the uptake of aminoglycoside in the cells (Allison et al., 2011). In another study, *cis*-2 decenoic acid (*cis*-DA), a fatty acid signaling molecule, was able to convert the persister cells to metabolically active form by increasing the protein synthesis rate and *cis*-DA was also able to enhance the antibiotic efficacy of ciprofloxacin against killing persister cells (Marques et al., 2014). It could also be important to consider antimicrobial approaches that physically or chemically disrupt cells rather than interfering with cellular processes (Hurdle et al., 2011).

In addition to therapeutics designed to target the metabolically dormant subpopulations, specific targeting of metabolically active subpopulations may be beneficial. For example, it is possible that targeting distinct “producer” biofilm subpopulations can promote the collapse of the biofilm in a similar manner that an overabundance of “cheaters” can result in community collapse (Wang et al., 2015; Ozkaya et al., 2018). Identification of “altruistic” biofilm community traits may yield insight into the development of efficacious therapeutics capable of disrupting biofilms via targeted eradication of shared resource-producing subpopulations. The production of community resources that are known to be differentially synthesized within specific biofilm populations dramatically impacts overall biofilm architecture and stability and therefore any population producing a community resource represents a promising therapeutic drug target. For example, in case of *P. aeruginosa* where siderophore production is required by the biofilm community to colonize an iron-limited environment like the eukaryotic tissue, targeting this public good that is not produced by the cheater population could be an attractive therapeutic target (Julou et al., 2013).

Another shared resource that can potentially be targeted is EPS production. By targeting EPS, we can promote dispersal of these recalcitrant communities. However, it is known that the cells dispersed from biofilms are physiologically distinct and more virulent compared to both their planktonic and biofilm counterparts (Chua et al., 2014). Therefore, triggering a biofilm community dispersal by targeting the EPS using the EPS-degrading enzymes can be associated with increased disease severity (Fleming and Rumbaugh, 2018). This microbial dissemination within the host can impose a major risk on healthcare-associated infections (Percival et al., 2015) and new ways to keep a check on the dispersal ability should therefore also be determined.

Additionally, while the bulk of this review focuses on the discussion of clonal biofilms, there are important mechanisms and shared resources that can be targeted in genetically diverse biofilms. For example, the horizontal exchange of genetic information is a major driver for the spread of multi-drug resistance among pathogens present in a biofilm. In *S. aureus*, the biofilm matrix and a close cell-to-cell contact provides a rich environment for the neighboring cells to undergo the processes of genetic transfer via both conjugation and mobilization, resulting in the horizontal spread of antibiotic resistance determinants (Savage et al., 2013). Biofilm formation therefore promotes the process of horizontal gene transfer by providing a privileged environment inducing the expression of natural competence in certain species (Molin and Tolker-Nielsen, 2003; Madsen et al., 2012). Since horizontal gene transfer via conjugation plays a vital role in the spreading of resistance genes within the biofilm community, antibiotics targeting bacterial conjugation can be a therapeutic target for biofilm eradication. However, combating horizontal gene spread in biofilm is subject to intense research and debate as plasmid transfer generally occurs in the outer metabolically active subpopulation and is subjected to availability of environmental factors like oxygen and nutrients (Stalder and Top, 2016). In general, a more targeted approach impacting only a subset of cell populations will less likely result in disrupting the host microbiome (Becattini et al., 2016).

In addition to the strategies discussed above, combinatorial therapeutics can be used to target multiple subpopulations residing in a biofilm (Band et al., 2019). The use of a synthetic peptide along with different classes of antibiotic can help enhance the bactericidal efficacy of these antibiotics in clearing both gram-positive and gram-negative bacterial infections. The peptides can disrupt the stringent stress response in the bacteria resulting in a more relaxed bacterial state, which can be easily targeted by the antibiotic treatment (Pletzer et al., 2018). It has been shown that spatially distinct subpopulations of *P. aeruginosa* comprising both metabolically active cells and relatively inactive cell subpopulations behaved differently when exposed to different antimicrobial agents like colistin, ciprofloxacin, and tetracycline (Pamp et al., 2008). In this study, the metabolically dormant cells were shown to be tolerant to ciprofloxacin and tetracycline but susceptible to colistin whereas the opposite profile was observed for the metabolically active subpopulation. Therefore, while individual antibiotic treatment did not clear the entire biomass, eradication of the biofilm could be achieved with combined antimicrobial treatment (Pamp et al., 2008).

**FIGURE 3:**
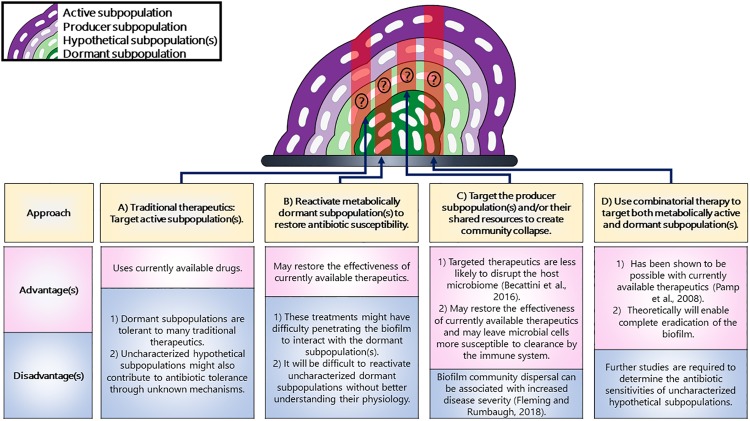
Strategies for targeting distinct subpopulations in a biofilm. **(A)** Traditional therapeutics are known to target active subpopulations but not dormant ones. **(B)** The metabolically dormant subpopulations that reside in the center can be reactivated to restore antibiotic susceptibility. **(C)** The subpopulations producing shared resources could result in the collapse of the community. **(D)** A combinatorial therapeutic approach could be used that could target both the active and dormant subpopulations. The populations potentially targeted by each strategy have been highlighted in red shading. Additionally, it is unknown whether or not uncharacterized hypothetical subpopulations would be targeted by these strategies and have therefore been marked with a question mark.

The strategies discussed in this review could be used for targeting biofilm subpopulation, with each one of them having their own advantages and disadvantages (Figure 3). Overall, we can use these strategies for exploiting biofilm subpopulations and we believe that using these strategies for targeting the distinct subpopulations having distinct genetic requirements could lead to the collapse of the entire biofilm.

## Concluding Remarks

Chronic microbial infections are often associated with biofilm communities. Biofilms promote microbial survival in the presence of environmental stressors such as exposure to antibiotics and host immune response (Donlan, 2002). Therefore, it is important that we understand the function and physiology of these microbial structures and identify weaknesses within the biofilm armor. The different subpopulations of cells present within the biofilm have defined roles to promote the overall stability and integrity of the biofilm ranging from promoting survival of the individual microbial cell to promoting the survival of the whole clonal community via shared resource production. The presence of both the selfish and altruistic traits within the biofilm subpopulation can, therefore, act to protect the entire community. Emergent technologies in the field of biofilm research is aiding in the discovery of these subpopulations and many more novel populations are expected to be identified. They could help decipher the core metabolic functions of biofilm subpopulations and their role within the context of community will enable the future development of antibiotics targeting these problematic microbial structures. Future work on defining the community functions of microbial subpopulations will reveal the metabolic susceptibilities of these communities, which could then be exploited for subsequent therapeutic targeting.

## Author Contributions

KB and CW conceived the research focus for the review article. KB drafted the manuscript, figures, and tables with feedback from CW. CW reviewed and edited the manuscript.

## Conflict of Interest Statement

The authors declare that the research was conducted in the absence of any commercial or financial relationships that could be construed as a potential conflict of interest.
